# Therapeutic Potential of Ginsenosides on Bone Metabolism: A Review of Osteoporosis, Periodontal Disease and Osteoarthritis

**DOI:** 10.3390/ijms25115828

**Published:** 2024-05-27

**Authors:** Seon-Yle Ko

**Affiliations:** Department of Oral Biochemistry and Institute of Dental Science, College of Dentistry, Dankook University, Cheonan 31116, Republic of Korea; seonyleko@dankook.ac.kr; Tel.: +82-41-550-1934

**Keywords:** ginsenoside, ginseng, osteoporosis, periodontal disease, osteoarthritis, osteoblast, osteoclast, periodontal ligament fibroblast, chondrocyte

## Abstract

Ginsenosides, bioactive compounds from the genus *Panax*, have potential therapeutic effects on diverse ailments, including diabetes. Emerging evidence suggests their involvement in bone metabolism. The present review summarizes the current understanding of the effects of ginsenosides on osteoporosis, periodontal disease, and osteoarthritis. Their mechanisms of action include effects on osteoblasts, osteoclasts, periodontal ligament fibroblasts (PDLFs), and chondrocytes, which are pivotal in maintaining bone, periodontal tissue, and cartilage homeostasis. Ginsenosides may exert their beneficial effects by enhancing PDLF and osteoblast activity, suppressing osteoclast function, augmenting chondrocyte synthesis in the cartilage matrix, and mitigating connective tissue degradation. Moreover, they possess antioxidant, anti-inflammatory, antimicrobial, and anti-pyroptotic properties. Their efficacy in increasing bone density, ameliorating periodontitis, and alleviating osteoarthritis symptoms has been demonstrated in preclinical studies using animal models. In terms of their mechanism of action, ginsenosides modulate cellular differentiation, activity, and key signaling pathway molecules, such as mitogen-activated protein kinases (MAPKs), while also regulating various mediators. Furthermore, the symptomatic relief observed in animal models lends further credence to their therapeutic utility. However, to translate these preclinical findings into clinical practice, rigorous animal and clinical investigations are imperative to ascertain the safety, efficacy, and optimal dosing regimens in human subjects.

## 1. Introduction

Bone is a highly calcified connective tissue that undergoes continuous remodeling orchestrated by a delicate balance between osteoclast-mediated bone resorption and osteoblast-mediated bone formation [1]. This remodeling is essential for skeletal formation, function, and mineral homeostasis. Disruption of this equilibrium because of various factors can lead to bone-related disorders, such as osteoporosis, periodontal disease, osteoarthritis, Paget’s disease, and multiple myeloma [2]. The prevalence of bone diseases, characterized by bone loss and compromised bone quality, is a significant health concern, particularly in the aging population [3].

Studies directed at treating bone-destructive diseases typically assess parameters such as osteoblast proliferation and activity, osteoclast production and resorption, and inflammatory responses implicated in bone degradation [4,5]. Evaluation often extends to the measurement of anti-inflammatory, antioxidant, and anti-pyroptotic activities, as well as the inhibition of extracellular matrix degradation [5,6,7]. Analyses of nuclear factor kappa-κB (NF-κB) and mitogen-activated protein kinase (MAPK) expression and activity in relevant cells serve to elucidate the signaling pathways involved [8,9,10,11]. Animal models of osteoporosis typically assess changes in bone density; in contrast, those of periodontal disease or osteoarthritis consider inflammatory mediators, connective tissue integrity, connective-tissue-degrading enzymes, and serum metabolic markers [12,13,14,15,16].

**Figure 1:**
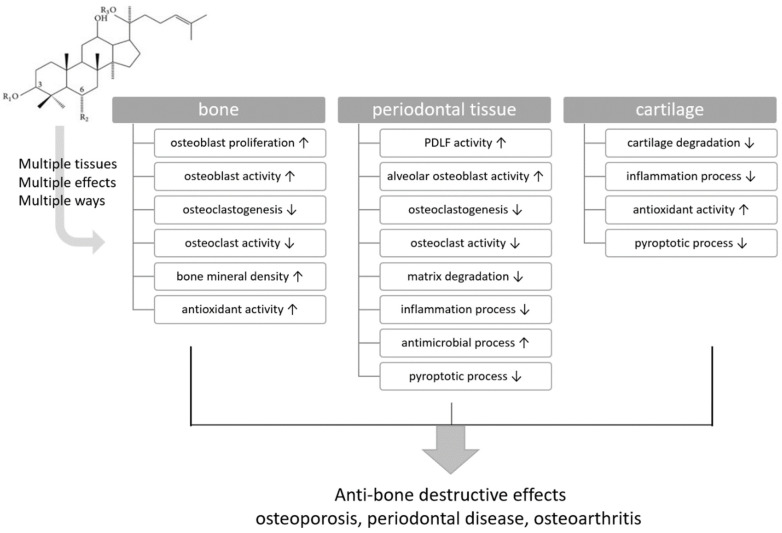
The anti-bone-destruction effect of ginsenosides can be explained by their effect on multiple tissues through different mechanisms. ↑: upregulation; ↓: downregulation.

**Figure 2:**
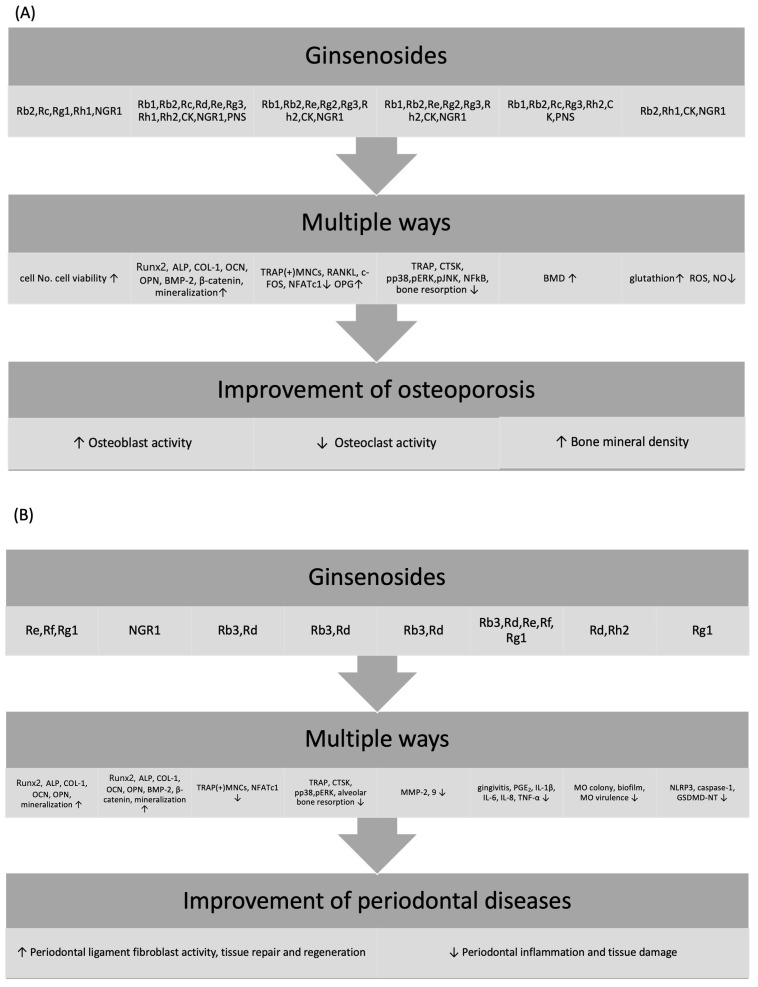

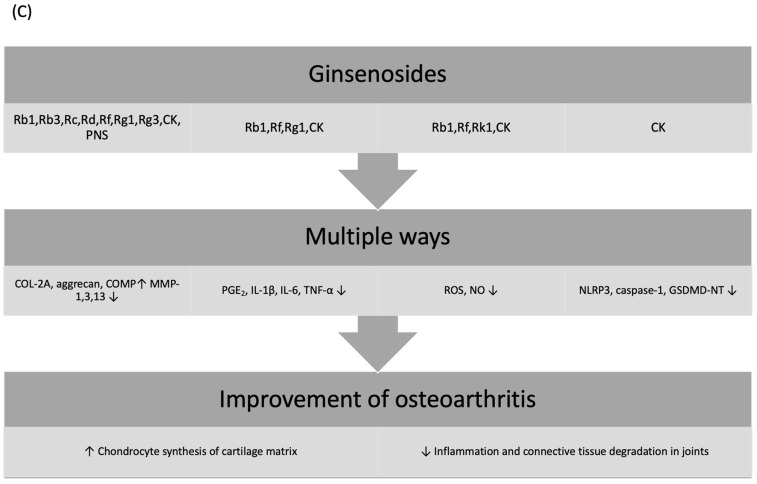
Effects and regulatory mechanisms of ginsenoside to alleviate (**A**) osteoporosis, (**B**) periodontal disease, and (**C**) osteoarthritis. ↑: upregulation; ↓: downregulation.

Osteoporosis, periodontal disease, and osteoarthritis have distinct pathogeneses. They all share the common feature of bone destruction. Numerous studies have explored treatments for these bone-destruction conditions, with the results of many of these studies demonstrating the potential of ginsenosides, the primary active compounds in ginseng, to mitigate bone destruction and inhibit cartilage and bone matrix degradation [17,18,19,20]. The present study is a narrative review of the literature that investigates the relationship between ginsenosides and bone-destructive diseases. The study offers a structural classification of ginsenosides and an overview of their biological effects on various target tissues, including bone, periodontal tissue, and cartilage. The effects of ginsenosides on osteoblasts, osteoclasts, periodontal ligament fibroblasts (PDLFs), and chondrocytes in these tissues were elucidated, as depicted in Figure 1. Prior to detailing the specific effects of ginsenosides on bone-destructive diseases, their various effects and mechanisms of action in protecting target tissues from disease are summarized (Figure 2). Table 1 outlines the functions of ginsenosides on target tissues across all investigated diseases.

**Table 1:** Summary of the functions of ginsenoside on bone, periodontal tissue, and cartilage.

Target Tissues	Bone	Periodontal Tissue	Cartilage
Ginsenoside	Functions		
G-Rb1	osteoblast activity ↑		cartilage degradation ↓
	osteoclastogenesis ↓		inflammatory process ↓
	osteoclastic activity ↓		antioxidant activity ↑
	bone mineral density ↑		
G-Rb2	osteoblastic cell proliferation ↑		
	osteoblast activity ↑		
	osteoclastogenesis ↓		
	osteoclastic activity ↓		
	antioxidant activity ↑		
G-Rb3		osteoclastogenesis ↓	cartilage degradation ↓
		osteoclastic activity ↓	
		matrix degradation ↓	
		inflammatory process ↓	
		gingivitis ↓	
G-Rc	osteoblastic cell viability ↑		cartilage degradation ↓
	osteoblast activity ↑		
	bone mineral density ↑		
G-Rd	osteoblast activity ↑	osteoclastogenesis ↓	cartilage degradation ↓
		osteoclastic activity ↓	
		matrix degradation ↓	
		inflammatory process ↓	
		antimicrobial process ↑	
G-Re	osteoblast activity ↑	periodontal ligament fibroblast activity ↑	
	osteoclastogenesis ↓	inflammatory process ↓	
	osteoclastic activity ↓		
G-Rf		periodontal ligament fibroblast activity ↑	cartilage degradation ↓
		inflammatory process ↓	intestinal inflammatory process ↓
			antioxidant activity ↑
G-Rg1	osteogenic differentiation from BMSCs ↑	periodontal ligament fibroblast proliferation ↑	cartilage degradation ↓
	adipogenic differentiation from BMSCs ↓	periodontal ligament fibroblast activity ↑	
		inflammatory process ↓	
		pyroptotic process ↓	
G-Rg2	osteoclastogenesis ↓		
	osteoclastic activity ↓		
G-Rg3	osteoblast activity ↑		cartilage degradation ↓
	osteoclastogenesis ↓		
	osteoclastic activity ↓		
	bone mineral density ↑		
G-Rh1	osteoblastic cell proliferation ↑		
	osteoblast activity ↑		
	antioxidant activity ↑		
G-Rh2	osteoblast activity ↑	antimicrobial process ↑	
	osteoclastogenesis ↓		
	osteoclastic activity ↓		
	bone mineral density ↑		
G-Rk1			inflammatory process ↓
CK	osteoblast activity ↑		cartilage degradation ↓
	osteoclastogenesis ↓		chondrocyte proliferation ↑
	osteoclastic activity ↓		chondrocyte differentiation ↑
	matrix degradation ↓		inflammatory process ↓
	bone mineral density ↑		pyroptotic process ↓
	antioxidant activity ↑		
NGR1	osteoblastic viability ↑	alveolar osteoblast activity ↑	
	osteoblastic differentiation ↑		
	osteoblast activity ↑		
	osteoclastogenesis ↓		
	osteoclastic activity ↓		
	antioxidant activity ↑		
PNS	osteoblast activity ↑		joint destruction ↓
	osteoclastogenesis ↓		inflammatory process ↓
	osteoclastic activity ↓		
	bone mineral density ↑		
Ginseng Extracts	osteoblast activity ↑	periodontal ligament fibroblast proliferation ↑	
	osteoclastogenesis ↓	periodontal ligament fibroblast activity ↑	
	bone mineral density ↑	osteoclastogenesis ↓	
		osteoclastic activity ↓	
		matrix degradation ↓	
		alveolar bone protection	
		inflammatory process ↓	
		antimicrobial process ↑	

↑: upregulation; ↓: downregulation.

## 2. *Panax Ginseng* and Ginsenoside

*Panax ginseng* Meyer, a perennial plant species in the Araliaceae family, has long been used in traditional herbal medicine as a health supplement to enhance body function and alleviate fatigue [21]. The pharmacological properties of ginseng extracts were initially reported in the 1950s [22], prompting extensive research on their traditional uses, chemical composition, and biological effects. Notably, ginseng and its extracts have demonstrated anti-inflammatory and antioxidant effects, offering relief from various conditions, including diabetes, hypertension, gastric ulcers, inflammatory diseases, and cancer [23,24,25,26,27]. Ginsenosides, the principal pharmacologically active constituents extracted from ginseng roots, have been found to be largely non-toxic to normal human cells. The results of recent studies have also implicated ginsenosides in the inhibition of bone resorption and the promotion of bone formation [5]. Ginsenosides are a widely used dietary supplement, with differing regulatory frameworks in different countries. In the United States, ginseng and its extracts, including ginsenosides, are generally recognized as safe (GRAS) for use in foods and dietary supplements. In South Korea, ginseng containing ginsenosides is approved as a health-functional food (HFF) by the Ministry of Food and Drug Safety (MFDS).

**Figure 3:**
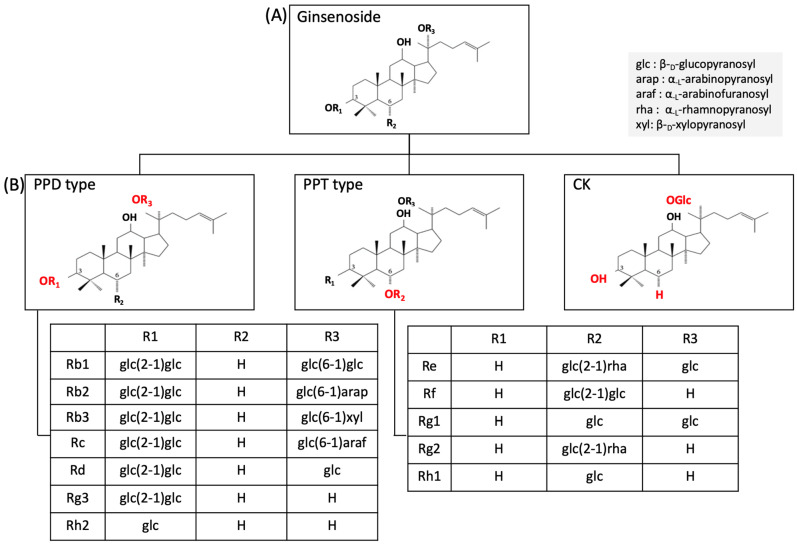
Types of ginsenosides. (**A**) Backbone structure of a ginsenoside, (**B**) structure of different types of ginsenosides with their side chains (R_1_, R_2_, and R_3_) in the PPD and PPT group. PPD, protopanaxadiol; PPT, protopanaxatriol; CK, ginsenoside compound K.

While ginseng comprises carbohydrates, alkaloids, amino acids, polypeptides, vitamins, and trace elements, its main active components are ginsenosides, which are steroid compounds extracted from its roots [28]. Ginsenosides are categorized into three groups: protopanaxadiols (PPDs), protopanaxatriols (PPTs), and oleanolic acids (OAs). PPDs encompass the ginsenosides Ra1, Ra2, Ra3, Rb1, Rb2, Rb3, Rc, Rd, Rg3, Rh2, Rs1, and Rs2 and notoginsenoside R (NGR)4. PPTs consist of ginsenosides Re, Rf, Rg1, Rg2, and Rh1 and NGR1. The OA category comprises only the ginsenoside Ro. Additionally, compound K (CK), a non-natural PPD ginsenoside with the structure of 20-O-β-D-glucopyranosyl-20(S)-protopanaxadiol, has emerged as an important metabolite detected in the bloodstream following the oral administration of ginsenosides Rb1, Rb2, or Rc. PPDs and PPTs represent the predominant ginsenoside groups distinguished by the position of the sugar molecule. While the PPD group has a sugar molecule attached to C-3 and/or C-20 of sapogenin to form an oxyglycoside, the PPT group has a sugar molecule attached to C-6 and/or C-20 of sapogenin to form an oxyglycoside [6] (Figure 3).

## 3. Effects of Ginsenoside on Osteoporosis

Bone, a dynamic tissue that undergoes constant remodeling, relies on the coordinated action of osteoblasts and osteoclasts [29]. Dysregulation of these cells can lead to bone metabolic disorders such as osteoporosis [30], which is characterized by decreased bone formation and increased resorption. Osteoporosis predisposes individuals to reduced bone mass, a compromised microstructure, and heightened fracture risk [31]. Osteoblasts, derived from mesenchymal stem cells, synthesize the organic bone matrix, including collagen, which is crucial for bone mineralization [32]. The activity and differentiation of osteoblasts are regulated by several key signaling pathways. Canonical Wnt signaling and bone morphogenetic protein (BMP) pathways are pivotal for osteoblast differentiation, with osteoblast-specific transcription factors, such as runt-related transcription factor 2 (Runx2), type 1 collagen (COL-1), osteocalcin (OCN), and osteopontin (OPN), serving as markers of activity. Disruption of these pathways can impair osteoblast function, leading to inadequate bone formation. Osteoclastogenesis, in contrast, involves the expression of differentiation factors, such as c-Fos, nuclear factor of activated T-cells c1 (NFATc1), receptor activator of nuclear factor-κB ligand (RANKL), and osteoprotegerin (OPG), which are critical for osteoclast differentiation. Ovariectomy (OVX) is a common method used to induce osteoporosis in animal models, followed by the administration of active substances and subsequent observation of changes in bone density after 4–8 weeks. This comprehensive model allows researchers to closely mimic the human condition of postmenopausal osteoporosis, thereby providing valuable insights into potential therapeutic interventions.

Numerous ginsenosides have demonstrated favorable effects on osteoporosis in various cellular and animal studies (Table 1 and Table 2). These effects include osteoblast proliferation and activity, osteoclastogenesis, osteoclast activity, antioxidant properties, and augmented bone mineral density in animal models (Figure 1 and Figure 2A). These diverse actions highlight the multifaceted role of ginsenosides in bone health, emphasizing their potential in enhancing bone formation and inhibiting bone resorption.

Eleven types of ginsenosides, including ginsenoside Rb1, Rb2, and Rc, have been confirmed to increase the expression levels of key markers in osteoblasts, such as Runx2, alkaline phosphatase (ALP), COL-1, OCN, OPN, BMP-2, and β-catenin, and mineralization [8,9,12,13,17,33,34,35,36,37,38,39,40,41,42,43,44,45,46,47,48,49,50,51,52,53,54,55]. Furthermore, eight ginsenosides, including ginsenosides Rb1 and Rg3, have been demonstrated to inhibit both osteoclast generation and activity [8,9,12,13,17,33,34,37,38,39,40,41,42,44,45,46,47,48,53,54,55]. This inhibitory effect was evidenced by a reduction in the number of tartrate-resistant acid phosphatase-positive multinucleated cells (TRAP (+) MNCs) and RANKL, c-Fos, and NFATc1 levels and an increase in OPG expression levels. Additionally, decreases in TRAP, cathepsin K, p-p38, p-extracellular signal-regulated kinase (ERK), p-c-Jun N-terminal kinase (JNK), and NF-κB levels and bone resorption have been observed. Moreover, the antioxidant effects were corroborated by an elevation in glutathione levels and a decrease in reactive oxygen species (ROS) and nitric oxide (NO) production by four types of ginsenosides, including ginsenosides Rb2 and CK (Figure 2A). The antioxidative properties of ginsenosides further underscore their therapeutic potential by protecting osteoblasts from oxidative stress, a known contributor to osteoporosis.

Ginsenosides Rb2, CK, and NGR1 exhibit enhanced osteoblast activity and reduced osteoclast activity and antioxidant activity (Table 2) [12,13,33,34,47,48,49,50,51,52]. In osteoporotic animal models, the ginsenosides Rb1, Rb2, Rc, Rg3, Rh2, CK, and Panax notoginseng saponins (PNS) have been shown to ameliorate bone volume and density [8,12,13,17,33,35,46,55]. NGR1 was also shown to inhibit calvarial osteolysis in a mouse model [50]; in contrast, Rg1 induces osteoblast differentiation and suppresses adipogenic differentiation [56]. Furthermore, ginseng extract upregulates Runx2 and BMP expression in osteoblasts, resulting in improved bone density [57,58].

**Figure 4:**
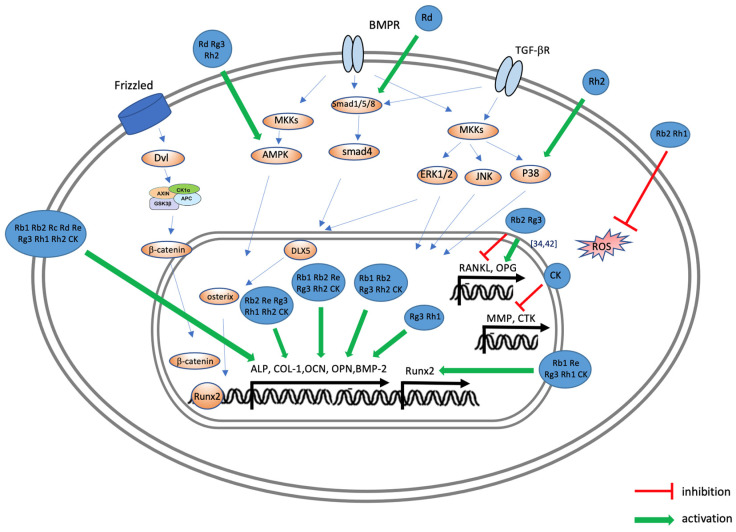
Possible molecular mechanisms of ginsenosides in inducing osteoblasts to improve the symptoms of osteoporosis and periodontal disease. In osteoblasts, BMP-2 binds to BMPR, activating the Smad complex (Smad 1, 5, and 8) or MAPK signaling pathways. This process in turn activates Runx2, promoting the expression of ALP, COL-1, OCN, OPN, and other proteins. Ginsenosides Rb1, Rb2, Rd, Re, Rg3, Rh1, Rh2, and CK promote the expression of key osteoblast markers (Runx2, ALP, COL-1, OCN, OPN, and BMP-2). Specifically, Rd enhances ALP, COL-1, and OCN expression via the Smad pathway, while Rh2 promotes COL-1 and OCN through p38 phosphorylation and regulates RANKL and OPG expression. Finally, Rb2 and Rh1 inhibit ROS secretion.

**Figure 5:**
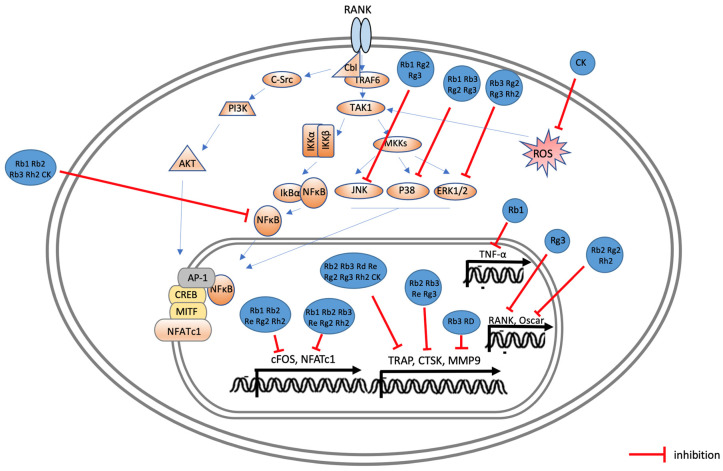
Possible molecular mechanisms of ginsenosides in inducing pre-osteoclasts to improve the symptoms of osteoporosis and periodontal disease. In osteoclasts, RANKL binds to RANK, which binds to TRAF6 intracellularly to regulate and activate downstream signaling pathways, including the NF-κB, INK, JNK, and p38 pathways. These pathways ultimately stimulate various transcription factors, such as the AP1 and NF-κB pathways, to promote osteoclast differentiation and bone resorption. Ginsenosides Rb1, Rg2, Rg3, and Rh2 inhibit osteoclast generation and activity by suppressing cFOS and NFATc1 through MAPK inhibition. Rb1, Rb2, Rb3, Rh2, and CK also inhibit osteoclast activity by suppressing NF-κB. Additionally, Rb3 and Rd inhibit MMP9 expression, and CK inhibits ROS secretion.

Figure 4 illustrates the effects of ginsenosides on the signaling pathways involved in osteoblast activation. When BMP-2 binds to BMPR in osteoblasts, it activates mothers against the decapentaplegic homolog (Smad) complex signaling pathway or the MAPK signaling pathway, which subsequently activates Runx2, a key osteoblast transcription factor, leading to the expression of various proteins including ALP [59]. The effects of ginsenosides on various pathways, such as the activation of p38 or AMP-activated protein kinase (AMPK) and the promotion of ALP, COL-1, OCN, and OPN expression, have been observed in various studies (Figure 4). This modulation of signaling pathways by ginsenosides suggests their capacity to enhance osteoblast differentiation and activity, critical processes for bone regeneration. Figure 5 illustrates the effects of ginsenosides on the signaling process during osteoclast generation and activity. When RANKL and RANK bind to osteoclast precursor cells, they regulate and activate downstream signaling pathways, such as the NF-κB and MAPK pathways, through TNF receptor-associated factor (TRAF)6, which is crucial for osteoclast differentiation. Ginsenosides inhibit the MAPK pathway and the NF-κB pathway, thereby suppressing the expression of transcription factors c-Fos and NFATc1, as well as the expression of TRAP, cathepsin K, and matrix metalloproteinases (MMPs) (Figure 5), thereby promoting osteoclast differentiation and bone resorption. The ability of ginsenosides to inhibit these critical pathways highlights their potential to effectively reduce bone resorption and maintain bone integrity.

Although the efficacy of ginsenosides has been assessed using various methods in cellular and animal models, categorizing the effects of different types or classes of ginsenosides remains a challenge. In animal models, only PPD types have been found to augment BMD activity; in contrast, correlations between the effects of PPD and PPT types have been elusive for most other functions. This complexity underscores the need for further research to fully understand the mechanisms and optimal applications of each ginsenoside type. In summary, ginsenosides have demonstrated the potential to improve bone density by enhancing osteoblast activity and inhibiting osteoclast activity, thus offering promise for mitigating bone deterioration associated with osteoporosis. Their multifaceted actions, including antioxidative and anti-resorptive effects, position ginsenosides as valuable candidates for developing new treatments for osteoporosis.

**Table 2:** Effects of ginsenosides on osteoporosis in cell line and animal studies.

Active Compound/Extracts	Properties	In Vitro Model	Activity and Mechanism	In Vivo Model	Activity and Mechanism
G-Rb1	osteogenic	isolated osteoblasts from DEX-OP rats	↑ ALP activity ↑ Runx2, OCN, and OPN mRNA (0.0145 mg/mL) [8]	**DEX-OP rats**	↑ BMD and BV/TV ↓ DEX-induced OP through the AHR/PRELP/NF-κB signaling ↑ AHR and PRELP proteins ↓ NF-κB p65 protein (IP 3 and 6 mg/kg/day) [8]
	anti-osteoclastogenic	RAW264.7 cells	↓ osteoclast differentiation ↓ TNFα mRNA ↓ c-Fos, NFATc1 mRNA ↓ nucleus translocation and activation of NF-κB ↓ JNK and p38 phosphorylation (0.1, 1, and 10 μM) [9]		
G-Rb2	osteogenic	MC3T3-E1 cells, H_2_O_2_-induced oxidative damage model	↑ cell proliferation ↑ ALP mRNA ↑ calcium deposition ↑ ALP, COL-1, OCN, and OPN mRNA against oxidative damage induced by H_2_O_2_ ↓ RANKL and IL-6 (0.1, 1, and 10 μM) [12]	**OVX-OP mice**	↓ blood MDA in OVX mice ↑ GSH activity in OVX mice ↑ BMD in OVX mice (IP 4.6 and 18.5 μmol/kg/day) [12]
				**KD-OP mice**	↑ bone volume fraction ↑ serum BALP ↑ OCN ↓ TRAP, PPAR-γ, and CTSK (IP 18.5 μmol/kg/day) [33]
	anti-osteoclastogenic	RAW264.7 cells	↓ TRAP (+) MNC generation and TRAP mRNA ↑ OPG mRNA ↓ bone resorption ↓ NFATc1, c-Fos, OSCAR, CTSK mRNA ↓ NF-κB activation ↓ STAT3 activation (0.1, 1, and 10 μM) [34]		
	antioxidant	MC3T3-E1, H_2_O_2_-induced oxidative damage model	↓ H_2_O_2_-induced production of ROS ↑ ALP, COL-1, OCN, and OPN mRNA against oxidative damage induced by H_2_O_2_ (0.1,1,10 μM) [12]		
G-Rc	osteogenic	MC3T3-E1 cells	↑ cell viability ↑ ALP staining ↑ calcium deposition ↑ β-catenin, p-GSK-3β, Runx2, ALP, and COL-1 mRNA (25, 50, 100, 200, 400, and 800 μM) [35]	**OVX-OP mice**	↑ BMD ↑ trabecular bone number ↑ microstructure of trabecular bone ↑ Runx2, ALP, COL-1, BMP-2, OCN, mRNA, protein (gavage 25 and 50 mg/kg) [35]
G-Rd	osteogenic	MC3T3-E1 cells	↑ ALP, COL-1, OCN, OPN, and OSX mRNA ↑ BMP-2 mRNA ↑ calcium deposition ↑ AMPK ↑ Smad1/5 phosphorylation (10, 20, and 40 μM) [36]		
G-Re	osteogenic	MC3T3-E1 cells	↑ ALP activity ↑ Runx2, ALP, COL-1, OCN, and mRNA ↑ calcium deposition (5, 10, 25, 50, and 100 μM) [37]		
	anti-osteoclastogenic	BMMs	↓ TRAP (+) MNCs generation ↓ TRAP activity ↓ NFATc1, c-Fos, and TRAP mRNA ↓ ERK phosphorylation (1, 2.5, 5, 10, 25, 50, and 100 μM) [38]	**zebrafish model**	more narrow distribution of TRAP staining ↓ TRAP and CTSK mRNA [38]
G-Rg1	osteogenic	BMSCs	↑ osteogenic differentiation of BMMSCs (5,10,20 μg/mL) [56]		
	antioxidant	BMSCs	↓ adipogenic differentiation by decreasing oxidative stress ↓ adipocyte distribution aging mice (5, 10, 20 μg/mL) [56]		
G-Rg2	anti-osteoclastogenic	BMMs	↓ osteoclast differentiation ↓ c-Fos and NFATc1 mRNA ↓ TRAP, Acp5, and Oscar mRNA ↓ p38, ERK, and JNK phosphorylation (1, 5, 10, 20, and 40 μM) [39]		
G-Rg3	osteogenic	MC3T3-E1 cells	↑ phosphorylated AMPK and autophagy ↑ Runx2, ALP, COL-1, OCN, and OPN mRNA ↑ calcium deposition ↓ mTOR signaling (10 and 20 μmol/L) [17]	**OVX-OP mice**	↓ OVX-induced BW increases, BMD decreases, and histological changes in femur tissues ↑ Runx2, ALP, COL-1, OCN, and OPN ↓ TRAP ↑ autophagy and AMPK signaling ↓ mTOR signaling (IP 20 mg/kg) [17]
		MC3T3-E1 cells	↑ ALP, COL-1 mRNA (10 and 100 μg/mL) [41]		
		Primary osteoblasts	↑ ALP activity ↑ calcium deposition ↓ RANKL mRNA and protein ↑ OPG mRNA and protein (1, 5, 10, 20, and 100 μM) [42]	**GC-OP**	↓ DEXA-induced BW increases and BMD decreases ↓ TRACP-5b activity ↓ CTx ↑ BMP-2, BMPR1A, and Runx2 mRNA (gavage 10 and 20 mg/kg) [42]
	anti-osteoclastogenic	RAW264.7 cells	↓ pit formation ↓ TRAP (+) MNC generation ↓ RANK, TRAP, and CTSK mRNA ↓ p38, ERK, and JNK phosphorylation (0.01, 0.1, 1, 10, and 100 μM) [40]		
G-Rh1	osteogenic	MC3T3-E1 cells	↑ cell growth ↑ ALP activity and COL-1 protein ↑ calcium deposition ↑ BMP-2 and Runx2 mRNA (0.01, 0.05, 0.5, and 5 μg/mL) [43]		
	antioxidant	AMA presented MC3T3-E1 cells	↑ glutathione ↓ ROS production enhanced by AMA (0.01, 0.05, 0.5, and 5 μg/mL) [43]		
G-Rh2	osteogenic	MC3T3-E1 cells	↑ ALP, COL-1, OCN, and OSX mRNA ↑ calcium deposition ↑ AMPK phosphorylation ↑ p38 phosphorylation [44]	**C57BL/6 mice**	↑ BMD (IP 3 mg/kg) [46]
		MC3T3-E1 cells	↑ ALP, COL-1 OCN, OPN, and OSX mRNA ↑ calcium deposition ↑ PKD and AMPK phosphorylation [45]		
	anti-osteoclastogenic	BMMs	↓ TRAP (+) MNC generation ↓ c-Fos, NFATc1, TRAP, and Oscar ↓ ERK phosphorylation ↓ NF-κB (5, 10, and 20 μM) [46]		
CK	osteogenic	MC3T3-E1	↑ ALP activity ↑ Runx2, ALP, COL-1, mRNA ↑ OPG mRNA ↑ Wnt10b, Wnt11, Lrp5, β-catenin (1, 2, 4, 8, and 16 μM) [47]	**rat open femoral fracture model**	↑ fracture repair (local injection 500 μM) [48]
				**OVX-OP mice**	↓ osteoclast number and surface area ↑ bone structure characteristics ↑ ALP, OCN, and OPN (IHC staining) ↓ MMP-9 and CTSK (IHC staining) (IP, 10 mg/kg) [13]
		BMSCs	↑ ALP, OCN, OPN, and OSX mRNA ↑ calcium deposition ↑ nuclear translocation of β-catenin, expression of Runx2 ↑ hUVEC formation (1 and 10 μM) [48]		
	anti-osteoclastogenic	RAW264.7 cells, BMMs	↓ TRAP (+) MNC generation ↓ NF-κB phosphorylation ↓ bone resorption (1 and 10 μM) [13]		
	antioxidant	RAW264.7 cells	↓ ROS activity (1 and 10 μM) [13]		
NGR1	osteogenic	hASCs	↑ cell migration and osteogenic differentiation ↑ VEGF mRNA ↑ adhesion and spreading of hASCs on the bio-inert glass surface ↓ RANKL/OPG expression ratio (0.01, 0.05, 0.5, and 5 μg/mL) [49]		
		MC3T3-E1 cells	↑ ALP activity ↑ ALP, COL-1, and OCN mRNA ↑ calcium deposition (5, 50, 100, 200, and 1000 μg/mL) [51]		
		MC3T3-E1 cells	↑ Runx2, ALP, and COL-1, OCN ↑ calcium deposition in OS injury model (10, 25, and 50 μM) [52]		
	anti-osteoclastogenic	Raw264.7 cells	↓ p38, ERK1/2, JNK1/2, and NF-κB phosphorylation ↓ TRAP (+) MNC generation ↓ osteoclast bone resorption (5, 10, and 20 μM) [50]	**mouse calvarial osteolysis model**	↓ mouse calvarial osteolysis (IP 10 and20 mg/kg) [50]
	antioxidant	MC3T3-E1, H_2_O_2_-induced oxidative damage model	↓ H_2_O_2_-induced osteoblast apoptosis ↑ osteoblast viability ↓ H_2_O_2_-induced mitochondrial ROS restored mitochondrial membrane potential and blocked JNK activated by H_2_O_2_ (10, 25, and 50 μM) [52]		
PNS	osteogenic	MC3T3-E1 cells	↑ ALP activity and calcium deposition ↑ COL-1 and OCN mRNA (0.05 and 0.5 mg/mL) [53]		
		BMSCs	↑ ALP activity and calcium deposition ↑ ALP, Cbfa1, and bone sialoprotein mRNA ↑ p38 and ERK phosphorylation [54]		
		**OVX-OP mice**	↑ restore bone mass ↑ CD31 and OCN ↓ serum NTX (P.O. 40 and 80 mg/kg) [55]		
Ginseng extracts	osteogenic	MC3T3-E1 cells	↓ caspase-3 and -9 mRNA ↑ Bcl2, IAPs, and XIAP mRNA ↑ Runx2, ALP, and BMP mRNA ↑ ALP activity ↑ AKT phosphorylation ↓ JNK phosphorylation (250, 500, and 1000 mg/mL) [57]	**OVX-OP mice**	Pg or Bo alone did not affect OVX-induced bone loss recovered bone weight (Pg:Bo) ↑ BMD (Pg:Bo = 3:1) ↓ OC formation (Pg:Bo = 3:1) ↓ blood glucose level (Pg:Bo = 3:1) (P.O. 500 mg/kg/day) [58]
				**GC-OP mice**	↓ bone loss (P.O. 100 mg/kg/d or 500 mg/kg/d) [57]

↑: upregulation; ↓: downregulation; bold font: in vivo experiment.

## 4. Effects of Ginsenosides on Periodontal Disease

Periodontal disease, a chronic inflammatory condition instigated by oral bacteria, precipitates the progressive deterioration of tissues encompassing periodontal ligaments, connective tissue, and alveolar bone, potentially culminating in tooth loss [60,61]. This destructive process arises from the multifaceted interplay between biofilm-forming pathogenic bacteria and the host immune response [62]. Notably, more than 300 bacterial species, including *P. gingivalis*, *P. intermedia*, and *A. actinomycetemcomitans*, have been implicated in the pathogenesis of periodontal disease, with their cell wall components and toxins inciting host immune responses and tissue destruction [63]. This interaction is mediated by cytokines and proteases secreted by host cells, such as neutrophils, mast cells, macrophages, and lymphocytes [62]. The intricate interplay between microbial pathogens and the host’s immune response underscores the complexity of periodontal disease, necessitating a multifaceted therapeutic approach.

The use of treatment modalities, such as scaling and periodontal surgery, and adjuncts, such as antibiotics and non-steroidal anti-inflammatory drugs, aims to mitigate periodontal disease by curbing bacterial proliferation and inflammation [64,65]. The efficacy of ginseng and its extracts in managing oral inflammatory disorders, including periodontal disease, evincing the inhibition of alveolar bone loss, and alterations in immune-related cytokines has been highlighted in recent studies (Table 3). These findings suggest that ginsenosides might offer an adjunctive therapy for periodontal disease.

The effect of ginsenosides on periodontal tissues spans various facets, including PDLF activity, osteoblast and osteoclast functions, connective tissue degradation, anti-inflammatory and antimicrobial responses, and anti-pyroptotic effects (Figure 1 and Figure 2B). PDLF, which is pivotal for periodontal ligament regeneration and activity, also contributes to alveolar bone remodeling by impeding epithelial cell and fibroblast apical migration from the gingiva and differentiation into osteoblasts or cementoblasts [66].

Ginsenosides Re, Rf, and Rg1 augment the expression of the transcription factor Runx2 and mineralization in PDLFs; NGR1, in contrast, increases alveolar osteoblast activity and mineralization [67,68,69,70,71]. Conversely, ginsenosides Rb3 and Rd suppress osteoclastogenesis and activity, abating alveolar bone resorption, and inhibiting matrix metalloproteinase activity in bone marrow-derived macrophages (BMMs) [10,72]. Moreover, ginsenosides Rb3, Rd, Re, Rf, and Rg1 curtail proinflammatory cytokine secretion and gingivitis severity, with Rd mitigating *P. gingivalis* virulence and biofilm formation [10,72]. Additionally, ginsenoside Rh2 exhibits antibacterial properties, restraining the growth of cariogenic agents, such as *S. mutans*, and curtailing *P. gingivalis* proliferation [73,74]. This antimicrobial action not only reduces the bacterial load but also disrupts the biofilm matrix, enhancing the efficacy of other therapeutic processes.

Furthermore, ginsenoside Rg1 mitigates pyroptosis by downregulating the NOD-like receptor family pyrin domain containing 3 (NLRP3), apoptosis-associated speck-like protein containing a caspase recruitment domain (ASC), caspase-1, and gasdermin D-NT (GSDMD-NT) in periodontal ligament cells [69]. Pyroptosis, a form of programmed cell death, orchestrated by the inflammasome, has been implicated in infectious diseases, with excessive pyroptosis exacerbating tissue damage [75,76]. Therefore, mitigating excessive pyroptosis may be beneficial for treating inflammation [77]. By reducing pyroptosis, ginsenosides not only preserve the viability of periodontal ligament cells but also alleviate the chronic inflammatory state characteristic of periodontal disease.

Ginseng extracts, in addition to their effects on cell proliferation and the expression of ALP and COL-1, have been shown to inhibit alveolar bone loss and MMP-9 expression in periodontal tissues, particularly in models of *P. gingivalis*-induced periodontal disease [78,79,80,81]. These findings indicate that ginseng extracts can modulate extracellular matrix remodeling processes, which are crucial for maintaining periodontal tissue architecture and function.

**Figure 6:**
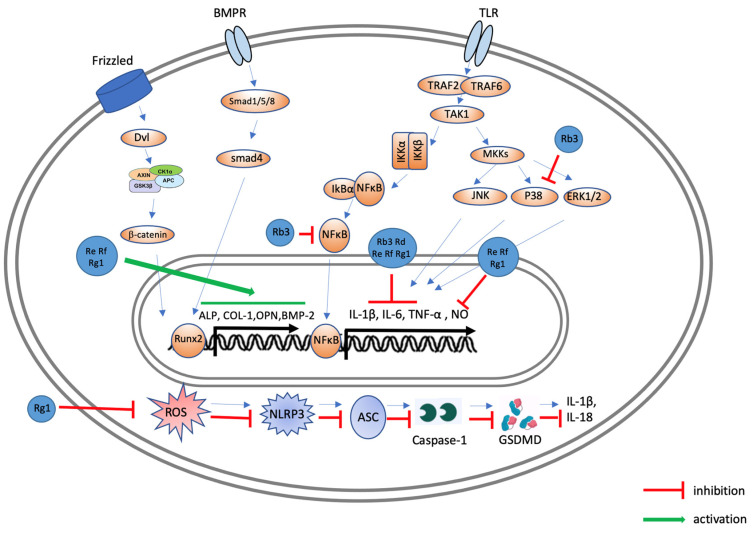
Possible molecular mechanisms of ginsenosides affecting periodontal ligament fibroblasts to improve periodontal disease. In PDLFs, BMP-2 binds to BMPR, activating the Smad complex or MAPK signaling pathways, activating Runx2. This process in turn promotes the expression of ALP, COL-1, OCN, OPN, and other proteins. Ginsenosides can promote the activity of various pathways. TRAF2/TRAF6 are activated by external signals, leading to the TAK1-mediated phosphorylation of MAPKs (JNK, p38, and ERK1/2). Phosphorylation of IκBα by IKK results in the release of p65/p50, inducing the transcription of proinflammatory genes such as *IL-*1β, *TNFα*, and *iNOS*. Ginsenoside Rb3 inhibits pro-inflammatory cytokines (IL-1β, IL-6, and TNF-α) by suppressing NF-κB or MAPK. Rg1 inhibits pyroptosis by reducing ROS production or NLRP3 expression. Re, Rf, and Rg1 enhance ALP, COL-1, OPN, and BMP-2 expression in periodontal ligament fibroblasts.

Figure 6 illustrates the effects of ginsenosides on signaling processes in PDLFs. Upon the binding of BMP-2 to the BMPR, the Smad complex (Smad 1, 5, and 8) signaling pathway is activated, subsequently promoting ALP expression through the activation of Runx2. Ginsenosides modulate various pathways within PDLFs, including the inhibition of p38 phosphorylation; the suppression of NF-κB activity; and increases in ALP, COL-1, OCN, and OPN expression levels. These pathways play pivotal roles in osteogenic differentiation and inflammation regulation, indicating that ginsenosides can effectively promote periodontal regeneration and reduce inflammatory damage. External signals stimulate TRAF2/TRAF6, leading to the transforming growth factor beta-activated kinase 1 (TAK1)-mediated phosphorylation of MAPKs (JNK, p38, and ERK1/2) and inhibitor of nuclear factor kappa-B kinase (IKK)α/IKKβ. The IKK-induced phosphorylation of the inhibitor of kappa B alpha (IκBα) results in its degradation and the release of p65/p50. The released p65/p50 translocates to the nucleus, repressing the transcription of genes such as interleukin (*IL*)-1β, tumor necrosis factor alpha (*TNFα*), and inducible nitric oxide synthase (*iNOS*). Ginsenosides exert inhibitory effects on NF-κB and MAPK signaling pathways, thereby dampening inflammatory responses (Figure 6). Figure 6 shows the pathways that lead to pyroptosis. Ginsenoside Rg1 decreases the expression levels of pivotal proteins implicated in this pathway, including NLRP3, ASC, caspase-1, and GSDMD-NT (Figure 6).

Distinct differences exist in the effects of PPD- and PPT-type ginsenosides on periodontal tissues. PPT-type ginsenosides chiefly enhance PDLF activity and exhibit anti-pyroptotic effects; PPD-type ginsenosides, in contrast, inhibit osteoclastogenesis, matrix degradation, and bacterial proliferation. Nonetheless, the precise effects of PPDs and PPTs on various cellular processes warrant further investigation.

In summary, the effects of various ginsenosides on periodontal tissues include the augmentation of PDLF and alveolar osteoblast activity, which are pivotal in periodontal ligament and alveolar bone remodeling. Additionally, ginsenosides impede osteoclastogenesis, thereby mitigating inflammation-induced alveolar bone destruction. Furthermore, they attenuate the activity of matrix-degrading enzymes implicated in connective tissue destruction, suppress cytokine-mediated inflammatory cascades, exhibit antibacterial effects by attenuating the virulence of periodontal pathogens, and demonstrate anti-pyroptotic activity. The multifaceted actions of ginsenosides not only address the symptoms but also the underlying causes of periodontal disease, offering a comprehensive therapeutic approach. Therefore, ginsenosides represent a potential therapeutic avenue for the management of periodontal disease and the amelioration of the tissue destruction associated with this condition.

**Table 3:** Effects of ginsenosides on periodontal disease in cell line and animal studies.

Active Compound/Extracts	Properties	In Vitro Model	Activity and Mechanism	In Vivo Model	Activity and Mechanism
G-Rb3	anti-osteoclastogenic	RAW264.7 cells and BMMs	↓ TRAP (+) MNC generation ↓ NFATc1, MMP-9, CTSK, and ACP mRNA ↓ MMP-9 and CTSK proteins ↓ p38, ERK, and p65 NF-κB phosphorylation (50, 100, and 150 μM) [10]	***P. gingivalis* -LPS-induced periodontitis in rats**	↓ p-ERK in alveolar bone surface, blood vessels, odontoblasts, and gingival epithelia ↓ gingivitis ↓ alveolar bone resorption (gingival injection, 100 μM) [10]
				***P. gingivalis -*LPS-induced periodontitis in rats**	↓ alveolar bone resorption ↓ TRAP (+) MNC generation (gingival injection, 100 μM) [14]
	anti-inflammatory/antimicrobial/anti-pyroptotic	***P. gingivalis****-*LPS*-*stimulated hPDLCs	↓ IL-1β, IL-6, and IL-8 mRNA ↓ p38 and p65 NF-κB, AKT phosphorylation (25, 50, and 100 μM) [14]		
G-Rd	anti-osteoclastogenic	RAW264.7 cells and BMMs	↓ TRAP (+) MNC generation ↓ RANKL-induced ACP, NFATc1, and MMP-9 mRNA (50 and 100 μM) [72]	**ligature-induced periodontitis in mouse**	↓ CEJ–ABC distances ↓ alveolar resorption (gingival injection 300 μM) [72]
	anti-inflammatory/antimicrobial/anti-pyroptotic	hGFs via LPS stimulation	↓ LPS-stimulated IL-1β, IL-6, and CXCL8 mRNA ↓ LPS-stimulated IL-1β, IL-6, and IL-8 secretion (100 and 200 μM) [72]	**ligature-induced periodontitis in mouse**	↓ bacteria colonies (gingival injection 300 μM) [72]
		***P. gingivalis***	↓ total biomass of bio films (100 and 200 μM) [72]		
G-Re, Ra8, Rf	osteogenic	hPDLCs	↑ calcium deposition ↑ Runx2, ALP, and OPN mRNA (40 μM) [67]		
	anti-inflammatory/antimicrobial/anti-pyroptotic	***P. gingivalis*** -LPS-stimulated hPDLCs	↑ HO-1 protein via the nuclear translocation of Nrf2 ↑ The HO-1 protein is regulated by EGFR ↓ PGE2, NO, and IL-6, TNF-α secretion ↓ COX2 and NOS protein (5, 10, 20, and 40 μM) [67]		
G-Rg1	osteogenic	hPDLCs	↑ cell proliferation ↑ Runx2, ALP, COL-1, OCN, and OPN mRNA ↑ calcium deposition (10 μmol/L) [68]		
		hDPSCs	↑ cell proliferation ↑ DSPP, ALP, and OCN mRNA ↑ BMP-2 and FGF-2 protein (5 μmol/L) [70]		
		hDPSCs	↑ cell proliferation ↑ ALP activity ↑ calcium deposition ↑ DSPP and DMP-1 mRNA (0.5, 2.5, 5, and 10 μmol/L) [18]		
	anti-inflammatory/antimicrobial/anti-pyroptotic	hPDLCs	↑ cell viability ↓ pyroptosis ↓ lactate dehydrogenase, IL-1β, and IL-18 secretion ↓ aberrant mitochondrial fission and mtROS production ↑ ATP content and mitochondrial membrane potential level ↑ Drp1 phosphorylation ↓ NLRP3, ASC, Caspase-1, and GSDMD-NT mRNA (50, 100, and 200 μM) [69]		
G-Rh2	anti-inflammatory/antimicrobial/anti-pyroptotic	***Streptococcus mutans, Streptococcus sobrinus, and Streptococcus sanguinis***	↓ biomass accumulation ↓ bacterial growth ↓ extracellular polysaccharide synthesis disrupts cell membranes ↓ acetaldehyde/alcohol dehydrogenase mRNA (6.25, 12.5, 25, 50, and100 ng μL^−1^) [73]		
		***P. gingivalis***	↑ clearance of P. gingivalis [74]		
NGR1	osteogenic	hAOBs	↑ ALP activity ↑ Runx2, OCN, and OPN ↓ p50 and p-p65 ↓ DKK1 mRNA ↑ AXIN2 and β-catenin mRNA ↑ calcium deposition (2.5, 5, 10, 20, and 40 μmol/L) [71]		
Ginseng extracts	osteogenic	hPDLCs	↑ Runx2, ALP, COL-1, and OPN mRNA protein ↑ Calcium deposition (50, 100, 150, and 200 μg/mL) [78]	**ligature-induced periodontitis in mouse** ***P. gingivalis* -LPS-induced periodontitis in rats**	ligature-induced periodontitis in mouse ↑ alveolar bone volume after tooth extraction ↑ BMD of the tooth socket P. gingivalis-LPS-induced periodontitis in rats ↓ alveolar bone loss restored BMD loss ↓ inflammatory invasion of periodontal cells (gingival injection 50 mg/kg) [78]
		hPDLCs	↑ cell proliferation (0.25 and 2 mg/mL) [79]	***P. gingivalis* -LPS-induced periodontitis in rats**	↓ alveolar bone loss ↓ MMP-9 around the gingival connective tissue (gingival injection 150, 300, and 360 mg/kg) [80]
	anti-osteoclastogenic	RAW264.7 cells	↓ LPS-stimulated TRAP(+) MNC generation (0.08, 0.4, and 2 mg/mL) [79]		
	anti-inflammatory/antimicrobial/anti-pyroptotic	***P. gingivalis*** -LPS-stimulated hPDLCs	↓ TNF-α, IL-1β, and IL-6 secretion ↓ PGE2 and NO secretion ↓ NOS and COX2 protein ↑ HO-1 protein (50, 100, 150, and 200 μg/mL) [78]		
		hPDLCs, RAW264.7 cells	↓ LPS-induced MMP-2 in PDLF ↓ LPS-stimulated activation of JNK and ERK in RAW264.7 cells ↓ LPS-stimulated degradation of IKB in RAW264.7 cells ↓ MMP-9 and iNOS in RAW264.7 cells ↓ NOS in RAW264.7 cells (0.08, 0.4, and 2 mg/mL) [79]		
		hGFs and hPDLCs	↓ TNF-α and IL-6 secretion (0.156, 0.312, and 0.625 mg/mL) [80]		
		***P. gingivalis***	Symphytum officinale (S), Panax Ginseng (G), and metronidazole (F) S+F: biofilm inhibition (98.7%) G+F: biofilm inhibition (98.2%) [81]		

↑: upregulation; ↓: downregulation; bold font: in vivo experiment; bold italic letters: bacterial strains.

## 5. Effects of Ginsenosides on Osteoarthritis

Osteoarthritis is a degenerative joint disease that causes pain and loss of joint function as a result of structural deformation resulting from the destruction of cartilage and basal bone [82]. Chondrocytes are responsible for maintaining the homeostasis of various matrix components in articular cartilage, making their role crucial during the progression of osteoarthritis [83]. Abnormal metabolic changes, such as inflammation, increased chondrocyte death, and extracellular matrix degradation, can lead to the development of osteoarthritis [84]. If harmful stimulation continues, the avascular cartilage has limited recovery ability, leading to chondrocyte pathology [85]. As osteoarthritis progresses, proteolytic enzymes cause the decomposition of cartilage matrix components, such as aggrecan and oligomeric matrix proteins, primarily in the cartilage. The pathological damage caused in osteoarthritis is typically regulated by signaling pathways, including Wnt/β-catenin, phosphoinositide 3-kinase (PI3K)/protein kinase B (AKT, PKB), and MAPK/NF-κB [7]. An in-depth understanding of these signaling pathways can provide valuable insights into novel therapeutic approaches, targeting specific molecular mechanisms to halt or reverse the progression of osteoarthritis. Currently, there is no effective treatment for degenerative osteoarthritis; however, nonsteroidal anti-inflammatory drugs are used to relieve pain [7]. Research is being conducted on the use of natural products that have traditionally been used for other purposes for the prevention and treatment of degenerative osteoarthritis [86].

The effects of ginsenosides on osteoarthritis, both in vitro and in vivo, have been mainly observed in terms of cartilage protection. In some studies, osteoarthritis has been induced using monoiodoacetate (MIA) to reproduce these conditions in animal models [16]. The effects of ginsenosides on articular cartilage tissue have been classified into four categories: the inhibition of matrix synthesis or the expression of matrix-degrading enzymes in chondrocytes, which play an important role in the destruction and regeneration of articular cartilage, and anti-inflammatory, antioxidant, and anti-pyroptotic activities (Figure 1 and Figure 2C). These multifaceted activities underscore the therapeutic potential of ginsenosides, suggesting that they might modulate various pathological processes simultaneously, thereby offering a comprehensive approach to osteoarthritis management.

In a number of studies, among 10 investigated ginsenosides, ginsenosides Rb1, Rb3, Rc, Rd, Rf, Rg1, Rg3, CK, and PNS showed chondroprotective effects in chondrocytes; however, Rk1 did not show these same effects [11,15,16,19,20,87,88,89,90,91,92,93,94]. The remaining ginsenosides, Rb1, Rb3, Rc, Rd, Rf, Rg1, Rg3, CK, and PNS, demonstrated cartilage protection by increasing the expression levels or synthesis of type 2 collagen (COL-2A) and aggrecan, while simultaneously inhibiting matrix-degrading enzymes, such as MMP-1, 3, and 13 (Table 4). Ginsenosides Rb3 and Rd significantly reduce the expression levels of MMP-3 in S12 murine articular cartilage cells [88]. Ginsenosides Rc, Rf, and Rg3 also significantly reduce MMP-13 expression levels in IL-1β-stimulated human osteosarcoma cells (SW1353 cells) [89]. Furthermore, ginsenosides Rb1, Rg1, and CK have been demonstrated to show in vitro effects and alleviate symptoms in animal models of osteoarthritis. Histopathological analysis has confirmed that ginsenoside Rb1 attenuates cartilage and glycosaminoglycan degradation in MIA-induced osteoarthritis [15,89]. Similarly, ginsenoside Rg1 was found to improve osteoarthritis symptoms by alleviating cartilage degradation and reducing MMP-13 in a rat model of osteoarthritis generated through anterior cruciate ligament transection (ACLT) [20]. CK significantly reduced the Osteoarthritis Research Society International (OARSI) score in a rat model of osteoarthritis induced by ligament incision and destabilization of the medial meniscus (DMM) [93]. Furthermore, CK-loaded hydrocaffeic-acid-conjugated chitosan patches were used to release CK from the cartilage defect site and inhibit cell death in osteoarthritic cartilage [19].

The activities of IKK, NF-kB, AKT, and p38 are reduced by five types of ginsenosides, namely Rb1, Rf, Rg1, Rk1, and CK. This process leads to a reduction in the production and serum levels of the pro-inflammatory mediators prostaglandin E_2_, IL-1β, IL-6, and TNF-α [15,20,87,90,92,93]. Moreover, ginsenosides Rb1, Rf, Rk1, and CK have been found to reduce intracellular ROS production or ROS and NO secretion [11,90,91,92]. Through research, it has been confirmed that different types of ginsenosides have anti-inflammatory and antioxidant effects. This is achieved by reducing the production of pro-inflammatory cytokines or ROS, which are associated with osteoarthritis. These anti-inflammatory and antioxidant effects are critical as they address both the symptoms and causes of osteoarthritis, potentially slowing disease progression. Furthermore, the effect of CK on pyroptosis, a type of programmed cell death caused by inflammasomes, has been confirmed in animal models of osteoarthritis induced by chondrocytes and MIA or DMM, as demonstrated by the inhibition of pyroptosis markers NLRP3, caspase-1, and GSDMD-NT [16,93].

In an animal model of MIA-induced postmenopausal arthritis in ovariectomized rats, the intra-articular administration of ginsenoside Rb1 increased BMP-2 and COL-2A expression levels [87]. In addition, treatment of MC3T3-E1 cells stimulated and cultured with H_2_O_2_ and CK resulted in significant increases in ALP activity, COL-1 content, and calcification, which are markers of osteoblast differentiation [92]. These results suggest that ginsenosides not only protect cartilage but also promote bone health, which is crucial for the overall management of osteoarthritis, particularly in postmenopausal women who are at higher risk of bone density loss.

**Figure 7:**
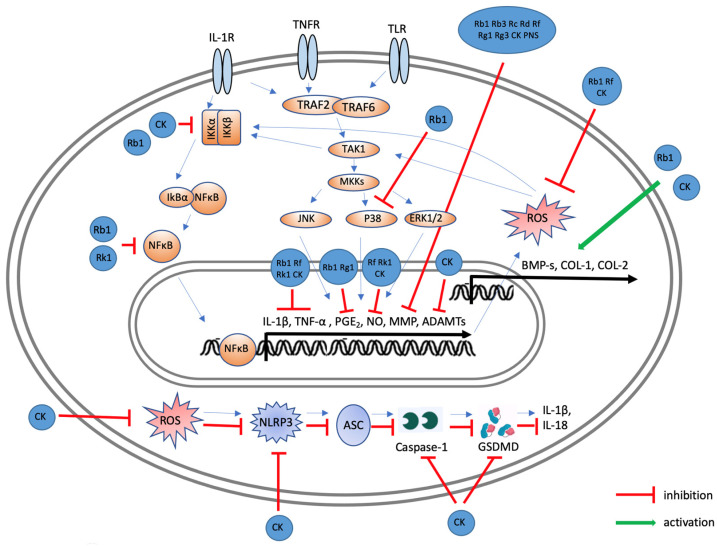
Possible molecular mechanisms of ginsenosides affecting chondrocytes to improve osteoarthritis. In chondrocytes, TRAF2/TRAF6 are activated by external signals, leading to the TAK1-mediated phosphorylation of MAPKs (JNK, p38, and ERK1/2). TAK1 and external signals also induce the phosphorylation of IKKα/IKKβ. The phosphorylation of IκBα by IKK leads to its degradation and release of p65/p50. This complex then enters the nucleus to regulate the transcription of target genes, including *IL-*1β, *TNFα*, *COX-2*, *iNOS*, *MMPs*, and *ADAMTS*. Ginsenoside Rb1 inhibits IL-1β and TNF-α secretion via p38 inhibition. Rk1 inhibits these cytokines through NF-κB suppression. Rb1, Rf, and CK inhibit ROS secretion, with CK specifically inhibiting pyroptosis by reducing NLRP3 expression.

Figure 7 shows the influence of ginsenosides on intracellular signaling pathways in chondrocytes triggered by external stimuli. Upon activation by external signals, TRAF2/TRAF6 initiate the TAK1-mediated phosphorylation of MAPKs (JNK, p38 MAPK, and ERK1/2). This cascade leads to the phosphorylation of IKKα/IKKβ. Subsequently, the phosphorylation of IκBα by IKK results in its degradation and the release of the p65/p50 complex. This complex translocates to the nucleus and represses the transcription of genes such as *IL-1β*, *TNF*α, cyclooxygenase (*COX)-2, iNOS*, *MMP*, and a disintegrin and metalloproteinase with thrombospondin motifs (*ADAMTS*) [7]. Ginsenosides inhibit the NF-κB and MAPK signaling pathways, thereby attenuating the inflammatory response. In addition, certain ginsenosides increase BMP-2 and COL-1 expression levels. Figure 7 outlines the signaling pathway of pyroptosis, a form of cell death initiated by the inflammasome. CK inhibits the production of ROS, a pivotal factor in pyroptosis, and suppresses the expression of NLRP3, as shown in Figure 7. The suppression of pyroptosis by CK highlights its role in preserving chondrocyte viability and function, which is essential for maintaining cartilage integrity and preventing the progression of osteoarthritis.

Among the ginsenosides investigated in the context of osteoarthritis, CK demonstrates a comprehensive array of effects, including cartilage protection; anti-inflammatory, antioxidant, and anti-pyroptotic effects; and the upregulation of osteoblast differentiation markers.

This section summarizes the activities of ginsenosides observed in cell experiments and animal models relevant to osteoarthritis. Although ginsenosides Rb1 and CK, both of which belong to the PPD type, exhibit diverse effects, no discernible correlation has been observed between the effects of PPD- and PPT-type ginsenosides in terms of cartilage protection and anti-inflammatory and antioxidant functions.

Various ginsenosides stimulate the synthesis of BMP-2, COL-1, and COL-2A by chondrocytes, thereby safeguarding the cartilage, inhibiting matrix-degrading enzymes involved in connective tissue degradation, and mitigating inflammation. Ginsenosides play a pivotal role in mitigating the degenerative processes associated with osteoarthritis by modulating cytokines and mediators of the inflammatory response and exhibiting anti-pyroptotic activity.

**Table 4:** Effects of ginsenosides on osteoarthritis and other inflammatory disease in cell line and animal studies.

Active Compound/Extracts	Chondroprotective		Anti-Inflammatory/Anti-Pyroptotic	
	Experimental Model	Activity and Mechanism	Experimental Model	Activity and Mechanism
G-Rb1	chondrocytes with osteoarthritis	↓ intracellular ROS production (30 and100 μg/kg) [11]		
	**hollow trephine on femur trochlea-induced rabbit OA**	↓ PGE_2_ and MMP-3 serum level ↑ TIMP-1 mRNA ↓ MMP-13-, MMP-3, and MMP-1 mRNA ↓ p-Akt, p-P65, and p-p38 protein ↓ chondrocyte-related irregularities (implant, 30 and 100 μg/kg) [11]		
	**MIA-induced OA**	↑ histological structure ↓ IL-1β, IL-6, and TNF-α in joint tissues ↓ miR-12-5p levels ↑ FGF-18 (gavage, 5 mg/kg) [15]		
	**MIA-induced OA in OVX rat**	↑ BMP-2 and COL-2A mRNA ↓ MMP-13, COX2, and TGF-β mRNA ↓ pathological changes in MIA-induced OA in OVX rats ↓ cartilage and GAG degradation (intraarticularly injection, 3 and 10 μg/kg) [87]	**MIA-induced OA in OVX rats**	↓ IL-1β, IL-6, MCP-1/CCL-2, COX2, and PGE_2_ serum level (intra-articular injection, 3 and 10 μg/kg) [87]
G-Rb3	S12 murine articular cartilage cell line	↓ MMP-3 secretion (1, 10, and 100 μg/mL) [88]		
G-Rc	chondrocyte (IL-1β-treated SW1353)	↓ MMP-13 secretion (5, 10, 15, and 20 μM) [89]		
G-Rd	S12 murine articular cartilage cell line	↓ MMP-3 secretion (1,10,100 μg/mL) [88]		
	chondrocyte (IL-1β-treated SW1353)	↓ MMP-13 secretion (5, 10, 15, and 20 μM) [89]		
G-Rf	chondrocyte (IL-1β-treated SW1353)	↓ MMP-13 secretion (5, 10, 15, and 20 μM) [89]	TNF-α-stimulated HT-29 cells, RAW264.7 cells	↓ IL-1β, IL-6, TNF-α, NO, and ROS secretion ↓ TNF-α/LPS-induced NF-κB phosphorylation [90]
G-Rg1	IL-1β-induced chondrocyte	↓ MMP-13, COX2, and PGE_2_ mRNA, protein ↓ COL-2 and aggrecan degradation (0.1, 1, and 10 μg/mL) [20]		
	**ACLT–OA rats**	↓ cartilage degeneration ↓ COL-2 loss and MMP-13 level (0.1, 1, and 10 μg/mL) [20]		
G-Rg3	chondrocyte (IL-1β-treated SW1353)	↓ MMP-13 secretion (5, 10, 15, and 20 μM) [89]		
G-Rk1			LPS-stimulated RAW264.7 cells	↓ NO, IL-6, IL-1β, TNF-α, and MCP-1 mRNA ↓ NF-κB and Jak2/STAT3 phosphorylation (10, 20, and 40 μmol/L) [91]
CK	H_2_O_2_-stimulated MC3T3-E1	↑ ALP and COL-1 activity ↑ calcium deposition ↑ ALP and COL-1 mRNA [92]	H_2_O_2_-stimulated MC3T3-E1 cells	↓ H_2_O_2_-induced ROS and NO ↓ IKK and IL-1β [92]
	PMCs	↓ MMP-3 and MMP-13, ADAMTS5 secretion ↓ IL-6 secretion ↓ IL-1β protein (10, 20, and 50 μM) [93]	PMCs	↓ NLRP3, GSDMD-NT, and caspase-1 protein (10, 20, and 50 μM) [93]
	immature murine articular chondrocytes (iMACs)	↑ chondrocyte proliferation ↑ chondrocyte differentiation ↓ cellular senescence and apoptosis-related gene expression [19]		
	chondrocytes	↓ MMP-3, MMP-13, ADAMTS4, and ADAMTS5 mRNA ↑ COL-2A mRNA ↓ IRE1α activation (0.3, 3, and 30 nM) [16]	chondrocytes	↓ Caspase-1, GSDMD protein (0.3,3,30 nM) [16]
	**destabilization of the medial meniscus (DMM) of mice**	↓ OARSI score ↑ COL-2 ↓ MMP-13 (diet supplement, 40 mg/kg) [93]	**destabilization of the medial meniscus (DMM) of mice**	↓ NLRP3 and GSDMD-NT protein 13 (diet supplement, 40 mg/kg) [93]
	**destabilization of the medial meniscus (DMM) of mice**	↑ aggrecan, COMP ↓ number of MMP-13-positive cells and TUNEL-positive cells ↓ number of pIkBα-positive cells ↓ AKT1, Annexin A2, and NFkB ↓apoptosis in osteoarthritic cartilage [19]		
	**MIA-induced rat OA**	↓ OARSI score ↓ MMP-13, IRE1α, and TXNIP level (gavage, 20 and 80 mg/kg/200 mL saline) [16]	**MIA-induced rat OA**	↓ IL-1β, IL-18, and TNF-α serum levels ↓ caspase-1 activity and NLRP3 level (gavage, 20 and 80 mg/kg/200 mL saline) [16]
PNS	**AIA rabbit**	↓ articular chondrocyte apoptosis ↓ lumbar vertebral and articular bone destruction ↓ arthritic muscular fiber atrophy ↓ inflammatory cell numbers ↑ bone density and microarchitecture (gavage, 75 mg/kg/day) [94]		

↑: upregulation; ↓: downregulation; bold font: in vivo experiment.

## 6. Conclusions

Ginsenosides have emerged as promising alternatives to conventional therapies for bone-destructive diseases, such as osteoporosis, periodontal disease, and osteoarthritis. This review highlights the efficacy of ginsenosides, the primary active constituents of ginseng, in addressing these conditions. The comprehensive nature of these natural compounds allows them to target multiple pathways involved in disease pathogenesis, offering a multifaceted approach to treatment. The tables and figures presented in this paper summarize the functions and mechanisms of action of various ginsenosides in impeding bone remodeling or destruction. Notably, several ginsenosides, including Rb1, Rb2, and CK, facilitate bone formation while simultaneously inhibiting bone resorption, which are crucial aspects of bone remodeling. In addition to bone remodeling, they exhibit antibacterial, anti-inflammatory, antioxidant, and anti-pyroptotic properties that can mitigate inflammation, oxidative stress, and inflammation-induced cell death. Owing to these multifaceted properties, ginsenosides hold the potential for use in the reconstruction of bone, periodontal tissue, and cartilage. Ginsenosides, such as Rb2, CK, and NGR1, demonstrate effects that augment osteoblast activity, diminish osteoclast activity, and confer antioxidant benefits. These actions collectively contribute to a favorable environment for bone healing and regeneration. Moreover, the ginsenoside Rg1 inhibits inflammasome-induced apoptosis in PDLFs. Among the ginsenosides studied in osteoarthritis treatment, CK has chondroprotective, anti-inflammatory, antioxidant, and anti-pyroptotic effects, and it increases the expression levels of osteoblast differentiation markers. These effects operate at the cellular level by modulating cell proliferation, differentiation, and activity. Ginsenosides regulate the expression of target pathway components, including MAPKs. At the molecular level, these factors influence the expression and secretion of various mediators.

The present review highlights the potential of ginsenosides for use in the management of bone-destruction diseases, particularly in alleviating inflammation by repressing pyroptosis. Recent research has shed light on the efficacy of ginsenosides Rg1 and CK in alleviating pyroptosis induced by inflammasomes. Further investigation is warranted to determine whether other ginsenosides exhibit similar effects.

This literature search revealed promising results from a variety of studies in cells and animals, but relatively few animal studies confirmed the results observed in vitro. For osteoporosis and periodontal disease, in vivo studies constitute only 35–38% of the research conducted compared to in vitro studies, while for osteoarthritis, in vivo studies constitute 58% of such research, which is a relatively higher percentage but still indicates a scarcity of animal studies overall. Additional animal studies and human clinical trials are therefore required to corroborate these outcomes.

Ginseng has long been used as a health supplement to promote overall well-being and has been the subject of clinical trials for the treatment of the common cold, diabetes, cardiovascular diseases, and cancer fatigue [95]. However, ginseng has low bioavailability, and the metabolites of ginsenosides produced in the body may impact its clinical efficacy. Further clinical research on the absorption, distribution, metabolism, and excretion of ginsenosides in humans is thus necessary.

The present review highlights the significance of ginsenosides in bone regeneration, extracellular matrix degradation, inflammatory responses, oxidative stress, and pyroptosis. Understanding the full spectrum of ginsenoside activities could lead to the development of comprehensive therapeutic strategies that address multiple aspects of bone-destructive diseases. Further research is required to comprehensively delineate the effects of ginsenosides on disease pathogenesis. Although studies on the effects of ginsenosides in the oral cavity have predominantly focused on PDLFs, additional investigations are warranted to explore their effects on other oral cavity cells as well as their potential synergistic effects with other drugs.

## Abbreviations

ABC: alveolar bone crest, ACLT: anterior cruciate ligament transection, ACP: acid phosphatase, ADAMTS: A disintegrin metalloproteinase with thrombospondin motifs, AHLs: acylated homoserine lactones, AHR: aryl hydrocarbon receptor, AIA: antigen-induced arthritis, ALP: alkaline phosphatase, AMA: antimycin A, AMPK: AMP-activated protein kinase, ARS: alizarin Red S, AXIN: BMD: bone mineral density, BALP: bone alkaline phosphatase, BMD: bone mineral density, BMMs: bone marrow-derived macrophages, BMP: bone morphogenetic protein, BMPR1A: BMP receptor 1A, BMSCs: bone marrow stromal cells, Bo: hot water extract of Brassicaoleracea, bPN: n-butanol extracts of PN, Cbfa1: core-binding factor a1, CEJ–ABC distances: distance from the cementoenamel junction(CEJ) to the alveolar bone crest(ABC), CK: compound K, COL-1: type I collagen, COL-2: type II collagen, COMP: cartilage oligomeric matrix protein, COX2: cyclooxygenase-2, CTSK: cathepsin K, DEX: dexamethasone, DEX-OP: dexamethasone-induced osteoporosis, DKK1: Dickkopf-related protein 1, DMP-1: dentine matrix protein-1, Drp1: dynamin-related protein1, DSPP: dentin sialophosphoprotein, EGFR: epidermal growth factor receptor, ERK: extracellular signal-regulated kinase, ERS: endoplasmic reticulum stress, FRGE: fermented red ginseng extract, GAG: glycosaminoglycans, gavage: intragastric administration, GC-OP: glucocorticoid-induced osteoporosis, GJIC: gap junction intercellular communication, GPCR84: G protein-coupled receptor, GSDMD-NT: Gasdermin D-N terminal, gavage: intragastric administration, GSH: reduced glutathione, hAOBs: human alveolar osteoblasts, hASCs: human adipose-derived stromal cells, hDPCs: human dental pulp cells, hDPSCs: human dental pulp stem cells, hGFs: human gingival fibroblasts, HO-1: hemeoxygenase1, hPDLCs: human periodontal ligament cells, HPU937 cells: human promonocytic U937 cells, hUVECs: human umbilical vein endothelial cells, IBD: inflammatory bowel disease, IHC: immunohistochemical, IL: interleukin, IP: intraperitoneal injection, IRE1α: inositol-requiring enzyme 1 alpha, IκB-α: NF-κB inhibitor alpha, Jak2: janus kinase2, JNK: c-Jun N-terminal kinase, KD: ketogenic diet, KD-OP: ketogenic diet-induced osteoporosis, KRGEs: Korean red ginseng extracts, LPS: lipopolysaccharide, MAPK: mitogen-activated protein kinase, MCP-1: monocyte chemotactic protein-1, M-CSF: macrophage colony-stimulating factor, MDA: malondialdehyde, MIA: monoiodoacetate, MMP: matrix metalloproteinase, mtROS: mitochondrial reactive oxygen species, NFATc1: nuclear factor of activated T-cells c1, NF-κB: nuclear factor-kB, NGR1: notoginsenoside R1, NLRP3: NLR family pyrin domain containing 3, NO: nitric oxide, NOS: nitric oxide synthase, Nrf2: nuclear factor-red blood cell 2-related factor2, OA: osteoarthritis, OARSI: Osteoarthritis Research Society International, OC: osteoclast, OCN: osteocalcin, OPG: osteoprotegerin, OPN: osteopontin, OS: oxidative stress, Oscar: osteoclast-associated receptor, OSX: osterix, OVX: ovariectomized, OVX-OP: ovariectomized induced osteoporosis, *P. gingivalis*: *Porphyromonas gingivalis*, P.O.: oral administration, Pg: 500 mg/kg/day P. ginseng extract, PGE_2_: prostaglandin E_2_, PGFE: Panax ginseng fruit extract, PKD: protein kinase D, PMCs: primary mouse chondrocytes, PNS: Panax notoginseng saponins, PPAR-γ: peroxisome proliferation-activated receptor γ, PRELP: proline/arginine-rich end leucine-rich repeat protein, RANKL: receptor activator of nuclear factor kappa B ligand, ROS: reactive oxygen species, Runx2: runt-related transcription factor2, STAT3: signal transducer and activator of transcription protein3, TNF-α: tumor necrosis factor-α, TRAP: tartrate-resistant acid phosphatase, TXNIP: thioredoxin interacting protein.
